# Arsenic Exposure and Predicted 10-Year Atherosclerotic Cardiovascular Risk Using the Pooled Cohort Equations in U.S. Hypertensive Adults

**DOI:** 10.3390/ijerph13111093

**Published:** 2016-11-07

**Authors:** Qingjiao Nong, Yiyi Zhang, Eliseo Guallar, Qiuan Zhong

**Affiliations:** 1 Department of Epidemiology, Guangxi Medical University School of Public Health, 22 Shuangyong Road, Nanning 530021, China; qjnong@163.com; 2 Departments of Epidemiology and Welch Center for Prevention, Epidemiology, and Clinical Research, Johns Hopkins Bloomberg School of Public Health, Baltimore, MD 21205, USA; yzhang74@jhu.edu (Y.Z.); eguallar@jhu.edu (E.G.)

**Keywords:** arsenic, cardiovascular disease, hypertension, risk assessment, gender difference

## Abstract

This study was to evaluate the association of urine arsenic with predicted 10-year atherosclerotic cardiovascular disease (ASCVD) risk in U.S. adults with hypertension. Cross-sectional analysis was conducted in 1570 hypertensive adults aged 40–79 years in the 2003–2012 National Health and Nutrition Examination Survey (NHANES) with determinations of urine arsenic. Predicted 10-year ASCVD risk was estimated by the Pooled Cohort Equations, developed by the American College of Cardiology/American Heart Association in 2013. For men, after adjustment for sociodemographic factors, urine dilution, ASCVD risk factors and organic arsenic intake from seafood, participants in the highest quartiles of urine arsenic had higher 10-year predicted ASCVD risk than in the lowest quartiles; the increases were 24% (95% confidence interval (CI): 2%, 53%) for total arsenic, 13% (95% CI: 2%, 25%) for dimethylarsinate and 22% (95% CI: 5%, 40%) for total arsenic minus arsenobetaine separately. For women, the corresponding increases were 5% (95% CI: −15%, 29%), 10% (95% CI: −8%, 30%) and 0% (95% CI: −15%, 19%), respectively. Arsenic exposure, even at low levels, may contribute to increased ASCVD risk in men with hypertension. Furthermore, our findings suggest that particular circumstances need urgently to be considered while elucidating cardiovascular effects of low inorganic arsenic levels.

## 1. Introduction

Arsenic contamination is a global environmental issue. Over 200 million persons worldwide are estimated to have been exposed at drinking water arsenic concentrations above the World Health Organization guideline level of 10 μg/L [1]. Even in the U.S., approximately 13 million Americans are under similar status of arsenic exposure [2]. In the past two decades, chronic exposure to inorganic arsenic (iAs) has caused increasing concerns for its cardiovascular toxicity. Evidence in cohort studies has shown that high levels of iAs in drinking water (>100 μg/L) are prospectively associated with increased cardiovascular outcomes, including carotid atherosclerosis [3], ischemic heart disease [4,5] and even a broad spectrum of heart diseases [6]. In contrast, the associations between low levels of iAs exposure (<100 μg/L in drinking water) and cardiovascular endings remain inconsistent, based on the available epidemiologic evidence [7]. Notably, the previous findings suggest that iAs exposure may act as a modifying instead of independent factor in the development of cardiovascular disease, at least under certain circumstances [7]. Therefore, the health effects of chronic exposure to low iAs levels on cardiovascular risk remain to be established, especially in a population with specific circumstances.

Hypertension, a well-known contributor to cardiovascular risk, is suggested to be associated with low levels of iAs involving not only long-term exposure, but also higher methylation capacity [8,9,10,11]. Indeed, relatively low levels of arsenic exposure have been shown to be positively associated with cardiovascular risk factors that are also highly related to hypertension, such as diabetes [12,13], triglyceridemia and cholesterolemia [13], circulating markers of endothelial dysfunction [14] and carotid intima-media thickness [15]. However, comprehensive cardiovascular risk derived from low levels of iAs is unclear yet among the hypertensive population.

The predicted 10-year risk of hard atherosclerotic cardiovascular disease (ASCVD), which uses the Pooled Cohort Equations developed by American College of Cardiology/American Heart Association (ACC/AHA) in 2013, represents the preclinical stage of comprehensive cardiovascular risk [16]. The Pooled Cohort Equations consider systolic blood pressure, including treated or untreated status, and permit sex- and race-specific estimates for African-American and non-Hispanic white men and women [16,17]; also, these equations have been proven to provide good calibration and discrimination [18].

U.S. adults in the National Health and Nutrition Examination Survey (NHANES) are typically exposed to low concentrations of arsenic from drinking water and food [19]. Thus, we analyzed the 2003–2012 NHANES data to evaluate the association of arsenic exposure, by measuring arsenic concentrations in urine, with predicted 10-year ASCVD risk in a representative sample of U.S. adults with hypertension. Urine arsenic compounds in NHANES samples mainly included iAs (arsenic and arsenate), non-toxic organic arsenic (arsenobetaine), as well as methylated species (monomethylarsonate (MMA) and dimethylarsinate (DMA)); DMA and arsenobetaine in urine were the major contributors to total urine arsenic concentrations. In contrast to arsenobetaine, which mainly reflects seafood-related organic arsenic exposure, urine DMA derives from not only the endogenous metabolic product of iAs, but also direct exposure to seafood consumption or other organic arsenicals [20,21]. Therefore, for urine arsenic used in this study, specific analytical strategies were performed to remove the underlying contribution of organic arsenic mainly from seafood.

## 2. Methods

### 2.1. Study Population

NHANES 2003–2012 were conducted by the U.S. National Center for Health Statistics to obtain a nationally-representative sample of the civilian noninstitutionalized U.S. population through a complex multistage sampling design. NHANES 2003–2012 randomly selected a one-third subsample including 13,577 participants aged 6 years and older for arsenic measurements. Among them, we excluded 475 participants missing total urine arsenic, 78 participants missing urine arsenic species of interest and 54 participants with total urine arsenic less than urine arsenobetaine. Then, we restricted the sample to 4993 participants aged 40–79 years, and subsequently excluded 667 participants with one of the following events, including heart failure history, coronary heart disease, angina, heart attack and stroke, 3 pregnant women, 2067 non-hypertensive participants, and 686 participants missing other variables of interest, leaving 1570 eligible participants for this study. The NHANES 2003–2012 study protocols were approved by the National Center for Health Statistics Institution review board (Protocol #98-12; Protocol #2005-06; Continuation of Protocol #2005-06; Protocol #2011-17). Oral and written informed consent was obtained from all participants.

### 2.2. Measures and Definitions

#### 2.2.1. Urine Arsenic Measurement

For arsenic analysis, spot urine samples were obtained during the physical examinations in arsenic-free containers, shipped on dry ice, stored at −70 °C or lower and measured within 3 weeks of collection at the Environmental Health Sciences Laboratory of the National Center for Environmental Health according to a standardized protocol [22,23,24,25,26,27]. The concentrations of total urine arsenic were determined by inductively-coupled-plasma dynamic reaction cell-mass spectrometry (ICP-DRC-MS) on a PerkinElmer ELAN^®^ 6100 DRC^Plus^ or ELAN^®^ DRC II (PerkinElmer SCIEX, Concord, ON, Canada). Urine arsenic species, including arsenite, arsenate, MMA, DMA and arsenobetaine, were analyzed by using high perform liquid chromatography (HPLC) coupled to an ICP-DRC-MS.

The limit of detection (LOD) for total urine arsenic was 0.6 μg/L for 2003–2004, 0.74 μg/L for 2005–2010 and 1.25 μg/L for 2011–2012. Furthermore, 1.65% of participants presented total urine arsenic levels below the LOD. In contrast, the percentages of participants with arsenite, arsenate and MMA levels below the LOD were 93.67%, 94.96% and 72.71%, respectively. The LODs for corresponding arsenic species were 1.2 and 0.48 μg/L, 1.0 and 0.87 μg/L and 0.9 and 0.89 μg/L in survey years 2003–2010 and 2011–2012, respectively. Consequently, these species were not used in our study. The LODs were 1.7 and 1.8 μg/L for DMA and 0.4 and 1.19 μg/L for arsenobetaine in survey years 2003–2010 and 2011–2012, respectively. The percentages of participants with levels below the LOD were 18.73% for DMA and 34.15% for arsenobetaine. Participants with urine arsenic levels under the LOD were assigned a level equal to the LOD divided by the square root of two. The inter-assay coefficients of variation for quality control pooled samples were 6.0% and 2.9% for lots with 5.34 and 11.91 μg/L mean total urine arsenic, 4.6% for lots with 5.67 μg/L mean DMA and 6.0% for lots with 5.86 μg/L mean arsenobetaine.

#### 2.2.2. Predicted 10-Year Risk of Hard ASCVD

Predicted 10-year risk of hard ASCVD for adults aged 40–79 years free from cardiovascular disease was calculated via using the sex- and race-specific Pooled Cohort Equations, which included the covariates of age, treated or untreated systolic blood pressure levels, total cholesterol and high-density lipoprotein cholesterol levels, current smoking status (yes, no) and diabetes status (yes, no). The equation form is expressed as:

Predicted 10-year risk of hard ASCVD = 1 − S_10_^exp(Individual sum−Mean sum)^ (1)

S_10_ is the baseline survival rate at 10 years. The individual sum is defined as the sum of “coefficient × value”; the values for continuous covariates are natural log-transformed; the values for interaction terms between age and lipids or age and systolic blood pressure are the product of the natural log of each covariate. The mean sum is overall mean “coefficient × value”. Sex- and race-specific values of S_10_, the coefficient, mean sum and algorithms for the equations were provided in detail elsewhere [16].

Information on age, sex, race/ethnicity, cigarette smoking, physician diagnosis of diabetes, as well as use of insulin, oral hypoglycemic medication and antihypertension medication was collected by a self-reported questionnaire. Race was categorized as non-Hispanic white, non-Hispanic black and other. During 2003–2006, total cholesterol and high-density lipoprotein cholesterol were measured using the Hitachi 704, 717 and 912. Beginning in 2007, the lipids were measured enzymatically in serum using the Roche Modular P chemistry analyzer [28,29]. Serum cotinine was measured by isotope-dilution HPLC/atmospheric pressure chemical ionization tandem mass spectrometry [30]. Current smoking was defined as serum cotinine ≥10 ng/mL, self-reported smoked at least 100 cigarettes in life and smoking now. Serum glucose concentration was determined by a hexokinase method. Diabetes was defined as a self-reported physician diagnosis, a self-reported use of insulin or oral hypoglycemic medication, a fasting serum glucose ≥126 mg/dL, or a non-fasting serum glucose ≥200 mg/dL.

#### 2.2.3. Hypertension

Blood pressure was measured up to four times. After discarding the first reading, mean systolic blood pressure and diastolic blood pressure levels were calculated, unless only one measurement was performed [10]. Hypertension was defined as a self-reported physician diagnosis, antihypertensive medication use, a mean systolic blood pressure ≥140 mmHg, or a mean diastolic blood pressure ≥90 mmHg.

#### 2.2.4. Other Variables

Information on education and seafood intake was collected by using a questionnaire. Seafood intake (fish, shellfish and/or mollusks, etc.) was identified based on the 24-h dietary recall interview and U.S. Department of Agriculture food codes. The food code details for identifying participant reports on seafood intake in the past 24 h were described elsewhere [31]. The body mass index (BMI) was calculated as weight in kilograms divided by height in meters squared. Urine creatinine was determined using the Jaffe reaction with a Beckman Synchron CX3 Clinical Analyzer (Beckman Instruments, Inc., Brea, CA, USA) before 2007 and an enzymatic method with a Roche/Hitachi Modular P Chemistry Analyzer (Roche Diagnostics Corp., Indianapolis, IN, USA) from 2007 [32].

### 2.3. Statistical Analysis

The Pooled Cohort Equations for predicted 10-year ASCVD risk were developed from sex-specific proportional-hazard models; hence, all analyses were stratified by men and women in this study. All statistical analyses were performed using the sampling survey commands in STATA Version 13.1 (StataCorp LP, College Station, TX, USA) to account for the complex sampling design, and spline functions were conducted in R version 3.2.5 (R Foundation for Statistical Computing, Vienna, Austria). In order to obtain unbiased point estimates and robust linearized standard errors, we used strata, primary sampling units and special sample weights for arsenic analyses in NHANES 2003–2012 [22]. Following the recommendations of the U.S. National Center for Health Statistics, new 10-year sample weights for NHANES 2003–2012 were created by dividing each 2-year subsample weight of arsenic by 5 [33]. The 2-sided statistical significance level was set at α = 0.05 for all statistical analyses.

Total urine arsenic and urine DMA were divided into quartiles based on the weighted distributions of corresponding arsenic concentrations in the study population. Predicted 10-year ASCVD risk was right-skewed and log-transformed for the analyses. We used linear regression models to estimate the ratio of geometric means in predicted 10-year ASCVD risk comparing quartiles 2–4 of total urine arsenic and urine DMA to the lowest quartile, respectively. *p*-values for the linear trend were obtained by including the medians for each urine arsenic quartile as continuous variables in the linear regression models. Furthermore, nonlinear dose-response relationships between urine arsenic levels and predicted 10-year ASCVD risk were estimated by using restricted quadratic splines with knots at the 10th, 50th and 90th percentiles of each urine arsenic distribution; *p*-values for nonlinear trend were obtained by Wald tests for restricted quadratic spline coefficients. Additionally, we conducted analyses to examine the presumption that the potential association of total arsenic or DMA with ASCVD risk derived from iAs exposure instead of organic arsenic. For this reason, we alternatively evaluated the association of total arsenic minus arsenobetaine, an indirect measure of urine iAs, with predicted 10-year ASCVD risk. Moreover, the association between urine arsenobetaine and predicted 10-year ASCVD risk was evaluated to identify whether organic arsenic had a potential effect on 10-year predicted ASCVD risk in this study. The above analytical strategies were consistent with total urine arsenic and urine DMA.

In this study, we used urine arsenic to assess iAs exposure based on the control of the organic arsenic contribution, which was generally reflected by arsenobetaine or seafood intake. Therefore, progressive models were adjusted to the potential confounding factors in regression models. First, we adjusted for age (continuous), race (non-Hispanic white, non-Hispanic black, other) and urine creatinine (log-transformed continuous). Then, we further adjusted for education (<high school, ≥high school), BMI (continuous), serum cotinine (log-transformed continuous), diabetes (yes, no), serum total cholesterol (continuous) and high-density lipoprotein cholesterol (continuous). Finally, we further adjusted for urine arsenobetaine (log-transformed continuous) and seafood intake (yes, no).

For sensitivity analyses, first, we assessed the nonlinear response odds ratios (ORs) for high 10-year predicted ASCVD risk by total arsenic, DMA and total arsenic minus arsenobetaine levels using restricted quadratic splines with knots at the 10th, 50th and 90th percentiles. Cutoffs for high 10-year predicted ASCVD risk were set at 7.5%, 10% and 15%, respectively. Second, we repeated the linear regression models for total arsenic, DMA and total arsenic minus arsenobetaine without adjusting for seafood intake. Third, the same analytical strategies were performed for non-hypertensive participants, as well as overall participants including hypertensive and non-hypertensive status.

## 3. Results

### 3.1. Baseline Characteristics of the Study Population

Among the hypertensive participants, the median of predicted 10-year ASCVD risk was 10.8% in men and 6.3% in women. The median total urine arsenic, urine DMA, urine arsenobetaine and total arsenic minus arsenobetaine concentrations were 9.2, 4.1, 1.9 and 6.7 μg/L, respectively, in men and 6.7, 3.2, 1.2 and 4.8 μg/L, respectively, in women. Overall, participants with higher total urine arsenic were more likely to be older men, non-Hispanic black women and to have higher urine creatinine and were less likely to be non-Hispanic white. Total urine arsenic concentrations were positively associated with urine DMA, total arsenic minus arsenobetaine and urine arsenobetaine in both men and women (Table 1 and Table 2).

### 3.2. Relationship between Urine Arsenic and 10-Year Predicted ASCVD Risk

**Figure 1:**
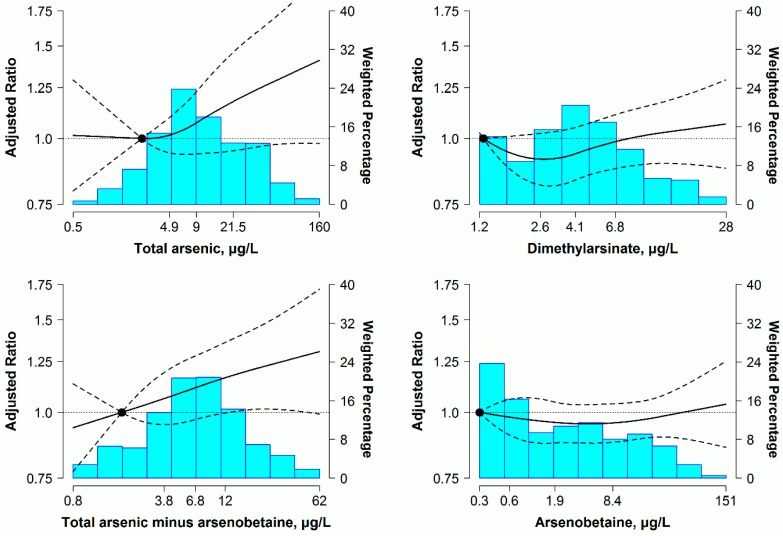
Ratio (95% confidence interval (CI)) of predicted 10-year atherosclerotic cardiovascular disease (ASCVD) risk by urine arsenic concentrations in hypertensive men (*n* = 739). Adjusted ratios (solid lines) and 95% CIs (curved dashed lines) were based on restricted quadratic splines for log-transformed urine arsenic concentrations with knots at the 10th, 50th and 90th percentiles. The reference value (circle) was set at the 10th of each arsenical distribution. Ratios (95% CIs) were adjusted for age (years), race (non-Hispanic white, non-Hispanic black, other), urine creatinine (log g/L), education (<high school, ≥high school), body mass index (kg/m^2^), serum cotinine (log ng/mL), diabetes (yes, no), total cholesterol (mg/dL), high-density lipoprotein cholesterol (mg/dL), arsenobetaine (log μg/L) and seafood (yes, no), except for the arsenobetaine model that was further adjusted for seafood only. Bars represent the weighted histogram of the urine arsenic distribution.

For men, non-significant associations of urine arsenic concentrations with predicted 10-year ASCVD risk were found in the initial models adjusted for age, race and urine creatinine (Table 3, Model 1). After full adjustment, higher total urine arsenic, urine DMA and total arsenic minus arsenobetaine levels, but not urine arsenobetaine, were associated with elevated 10-year predicted ASCVD risk. Participants in the highest quartiles of total urine arsenic, DMA and total arsenic minus arsenobetaine separately had 24% (95% confidence interval (CI): 2%, 53%), 13% (95% CI: 2%, 25%) and 22% (95% CI: 5%, 40%) higher 10-year predicted ASCVD risk than in the lowest quartiles, but a non-significant 2% (95% CI: −10%, 7%) lower 10-year predicted ASCVD risk for the corresponding comparison in arsenobetaine (Table 3, Model 3). Furthermore, increased trends in 10-year predicted ASCVD risk with increasing total arsenic, DMA and total arsenic minus arsenobetaine concentrations were found in dose-response analyses, with no significant departures from linearity (*p* > 0.05 for restricted quadratic spline coefficients). The ratios of predicted 10-year ASCVD risk presented an evident and persistent increase, especially at the higher concentration range for total arsenic, while responding to DMA concentrations with a gentle rise. In addition, a progressive association of ASCVD risk with total arsenic minus arsenobetaine was revealed throughout the range of arsenic concentrations (Figure 1).

**Figure 2:**
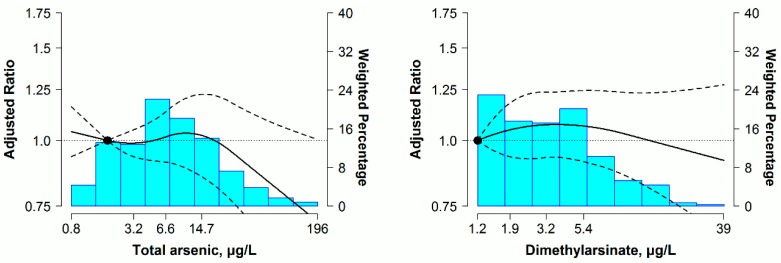

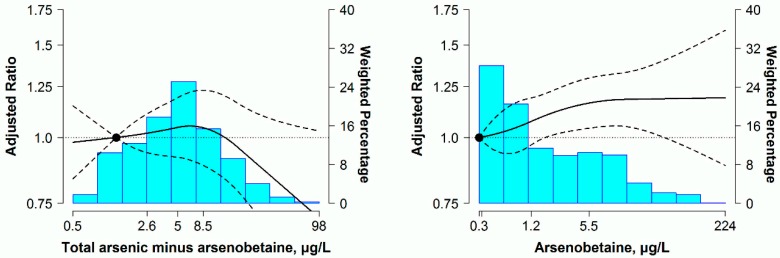
Ratio (95% CI) of predicted 10-year ASCVD risk by urine arsenic concentrations in hypertensive women (*n* = 831). Adjusted ratios (solid lines) and 95% CIs (curved dashed lines) were based on restricted quadratic splines for log-transformed urine arsenic concentrations with knots at the 10th, 50th and 90th percentiles. The reference value (circle) was set at the 10th of each arsenical distribution. Ratios (95% CIs) were adjusted for age (years), race (non-Hispanic white, non-Hispanic black, other), urine creatinine (log g/L), education (<high school, ≥high school), body mass index (kg/m^2^), serum cotinine (log ng/mL), diabetes (yes, no), total cholesterol (mg/dL), high-density lipoprotein cholesterol (mg/dL), arsenobetaine (log μg/L) and seafood (yes, no), except for the arsenobetaine model that was further adjusted for seafood only. Bars represent the weighted histogram of urine arsenic distribution.

For women, urine concentrations of arsenobetaine, except for total arsenic, DMA and total arsenic minus arsenobetaine, were significantly associated with 10-year predicted ASCVD risk. The fully adjusted geometric mean ratios for 10-year predicted ASCVD risk comparing the highest with the lowest quartiles of urine arsenic, were 1.05 (95% CI: 0.85, 1.29) for total arsenic, 1.10 (95% CI: 0.92, 1.30) for DMA, 1.00 (95% CI: 0.85, 1.19) for total arsenic minus arsenobetaine and 1.20 (95% CI: 1.05, 1.37) for arsenobetaine, respectively (Table 4, Model 3). No increased trends in 10-year predicted ASCVD risk were found across total arsenic, DMA and total arsenic minus arsenobetaine in dose-response analyses, but a slight increase was observed over the range of urine arsenobetaine concentrations, with no significant departure from linearity (*p* > 0.05 for restricted quadratic spline coefficients) (Figure 2).

### 3.3. Sensitivity Analysis

After adopting ORs for high 10-year predicted ASCVD risk regardless of ≥7.5%, ≥10% or ≥15%, the dose-response relationships were still similar to results plotted in Figure 1 and Figure 2 for total arsenic, DMA and total arsenic minus arsenobetaine (Figures S1 and S2). For the above three arsenic markers, linear regression analyses without adjustment for seafood intake still yielded similar findings in both men and women (Table S1). Moreover, for non-hypertensive participants and overall participants, the repeated analyses using the same analytical strategy showed non-significant associations between urine arsenic species and predicted 10-year ASCVD risk in men and women (Tables S2 and S3).

## 4. Discussion

In this national cross-sectional sample of U.S. adults, urine concentrations of total arsenic and DMA were positively associated with predicted 10-year ASCVD risk in men with hypertension. The significant associations were observed after adjustment for urine arsenobetaine and seafood intake, as well as confounding factors for ASCVD risk estimation. Furthermore, the associations persisted even using the total urine arsenic minus arsenobetaine. While no significant association was confirmed for urine arsenobetaine in men. For hypertensive women, no evidence supported positive associations of urine arsenicals except for arsenobetaine with predicted 10-year ASCVD risk. Overall, our findings suggest that low levels of iAs exposure may increase comprehensive cardiovascular risk with no apparent threshold in hypertensive men.

The positive associations between high levels of iAs exposure and cardiovascular outcomes were continuously observed in drinking water arsenic-enriched areas, whereas, the associations became non-significant in the low iAs levels [6,34,35,36]. For instance, a prospective study in Bangladesh reported that the hazard ratio for cardiovascular mortality comparing the highest arsenic level (≥300 μg/L) in drinking water with the lowest level (<10 μg/L) was 1.37 (95% CI: 1.07, 1.77), while for the low (10–49 μg/L) and low to moderate arsenic levels (50–149 μg/L), the corresponding hazard ratios were 1.03 (95% CI: 0.82, 1.29) and 1.16 (95% CI: 0.96, 1.40), respectively [36]. Indeed, current epidemiologic evidence in the U.S. tended toward non-significant association between low levels of arsenic from drinking water and cardiovascular outcomes [37,38]. A recent prospective research from the Strong Heart Study showed that chronic exposure to low to moderate arsenic levels, as measured in urine, was significantly associated with cardiovascular disease incidence and mortality, including coronary heart disease and stroke [39]; however, the study participants were enrolled from a sample with a high burden of diabetes and cardiovascular disease. In our analysis, a positive association between urine arsenic concentrations and predicted 10-year risk was confirmed in hypertensive men, although no significant relationship was observed for overall participants. This finding probably supports in part that iAs exposure at low levels is less likely to independently increase the cardiovascular risk for the general population.

The associations of low arsenic exposure levels with single cardiovascular risk factors have been reported by a number of studies. In a cross-sectional study from NHANES 2003–2004, total urine arsenic (median level 7.1 μg/L) was related to increased type 2 diabetes prevalence [12]. Another cross-sectional study in Mexico found significant associations of arsenic levels, including water arsenic (25.5 to 47.9 μg/L) and total urine arsenic (<55.8 μg/L), with diabetes and dyslipidemia [13]. Similarly, among 668 participants exposed to low arsenic levels in drinking water (median level of 23 μg/L) from Bangladesh, either well water or urine arsenic concentrations, were positively associated with plasma levels of soluble vascular adhesion molecule-1 [14]. Moreover, a series of cross-sectional studies concluded that low levels of arsenic exposure played a role in increasing carotid artery intima-media thickness [15,40]. In contrast, we evaluated the iAs-associated cardiovascular effect via using the predicted 10-year ASCVD risk, a measure for comprehensive cardiovascular risk in the preclinical stage, instead of a single risk factor. Therefore, our study could reflect iAs exposure at low levels as an ASCVD risk factor for the hypertensive population.

The potential mechanisms for iAs exposure in increased ASCVD risk are generally considered to be related to endothelial insufficiency and elevated systemic inflammation. Experimental evidence showed that 5 and 10 μmol/L of sodium arsenite increased reactive oxygen species (ROS) in human umbilical vein endothelial cells [41]. Furthermore, accumulation of ROS might produce a strong oxidant form, such as peroxynitrite, via coupling with nitric oxide (NO) [42]. Furthermore, sodium arsenite at 100 μg/L could directly suppress endothelial NO synthase activity and protein expression in vitro [43] and then decrease NO generation. Moreover, 10 ppm of sodium arsenite increased 3-nitrotyrosine, a biomarker for peroxynitrite, in innominate artery plaques of ApoE^−/−^/LDLr^−/−^ mice [44]. All of these iAs-related toxicity could attenuate NO bioavailability [45,46] resulting in endothelial insufficiency. For the systemic inflammation, increased interleukin-8 in vitro [47], elevated pro-inflammatory cytokine interleukin-6 [48], leukotriene E4 and prostacyclin in vivo [44] were involved in the biologic effects of iAs on ASCVD risk. In addition, other mechanisms for the increased ASCVD risk may be explained by evidence in vitro that sodium arsenite at 5 μmol/L could enhance the cellular uptake of oxidized low-density lipoprotein cholesterol [49].

Experimental studies have shown hypertension to deteriorate cardiovascular homeostasis through endothelial dysfunction and elevated inflammation response, which are the same as the main mechanisms for iAs-induced cardiovascular risk. For instance, aortic segments from hypertensive mice have lower levels of NO release than non-hypertensive mice [50]. Endothelial NO synthase phosphorylation and endothelial-dependent relaxation are significantly reduced in arteries from hypertensive mice [51,52]. Moreover, compared to non-hypertensive rats, heart and aorta from hypertensive rats have greater levels of pro-inflammatory cytokines, such as IL-1β, IL-6 and TNF-α [50,53], which can lead to cardiac injury and cardiac fibrosis [54,55]. On the other hand, the reduction of anti-inflammatory may contribute to the pathogenesis of cardiac injury in hypertensive animals [51]. Considering the closely similar mechanisms between iAs exposure and hypertension for ASCVD risk, thus, it may be one of the reasons why low levels of iAs exposure could enhance the ASCVD risk under hypertensive status. However, no animal model of hypertension has been identified yet for low-dose iAs with the above hypothesis; further research in vivo is needed to establish the triangle relationship among iAs concentrations, hypertension and ASCVD risk.

In our study, the significant associations between urine arsenic concentrations and predicted 10-year ASCVD risk were overwhelming in men, but not in women. Although the mechanisms underlying the gender differences are not well understood, vascular endothelial function, as the common target of iAs toxicity and hypertension, needs to be concerned in some evidence from animals or humans. Endothelial dysfunction has been suggested to be more severe in male than female rats with spontaneous hypertension [56]. In addition, male rats have grater superoxide anion production than female rats with both hypertension and non-hypertension; consequently, impaired NO bioavailability could be more evident in males than females [57,58]. Consistently, a study conducted in humans has reported that compared with hypertensive women, the levels of NO are significantly less in hypertensive men [59]. These findings suggest that although populations are concurrently exposed to iAs and hypertension even under similar conditions, men are more likely to develop the ASCVD risk that is mechanistically raised by endothelial dysfunction. Notably, a positive relationship between urine arsenobetaine and ASCVD risk was found in women, but not in men in this study, suggesting that the possibility in risk effect of organic arsenic exposure on ASCVD development cannot completely be ruled out for women. The differential associations in urine arsenobetaine also may contribute to the gender differences in ASCVD-related arsenic toxicity.

Some limitations in this study should be considered. First, we cannot establish the causal relation between urine arsenic concentrations and the increased ASCVD risk, owing to the cross-sectional design. However, the development of atherosclerosis seems to be less likely to intervene in the metabolism of arsenic compounds. Second, given the high percent of urine levels below the LODs for arsenite, arsenate and MMA, it is unknown to what extent the available urine arsenic represents iAs exposure in our analysis. Additionally, we cannot efficiently remove all potential contributions of organic arsenic derived from other foods or unknown sources. The association of urine arsenic with ASCVD risk could still be confounded by organic arsenic, although we have already adjusted for urine arsenobetaine and seafood intake in the regression models. Third, as a biomarker with a relatively short half-life, urine arsenic may not reflect chronic exposure, unless relatively constant and stable exposure from dietary pattern and drinking water can be ensured for study participants. Indeed, in terms of drinking water, arsenic exposure remains relatively stable over many years in the U.S. in absence of public health intervention [60,61]. Finally, the predicted 10-year ASCVD risk may be overestimated in our study, as the equations for non-Hispanic whites were used in other racial/ethnic participants according to the recommendation by ACC/AHA [16]. However, our results were not substantially modified even after excluding the participants of other race/ethnicity. Overall, our findings need to be interpreted with caution due to the above-mentioned limitations.

## 5. Conclusions

Positive associations of total arsenic, DMA and total arsenic minus arsenobetaine in urine with predicted 10-year ASCVD risk were observed among men with hypertension from a representative sample of U.S. adults. Further prospective research is needed to confirm the gender differences in the association between relevant arsenic levels and ASCVD risk in hypertensive population. As an extended study from high levels of arsenic exposure, our findings add to the concerns about the cardiovascular effect of chronic iAs exposure, even at relatively low levels, for an asymptomatic population with hypertension.

## Figures and Tables

**Table 1:** Baseline characteristics of the study population by quartiles of total arsenic concentrations in hypertensive men (*n* = 739).

Characteristics	Overall	Quartile 1	Quartile 2	Quartile 3	Quartile 4	*p* for Trend ^a^
		(≤4.92 μg/L)	(4.93–9.20 μg/L)	(9.21–21.56 μg/L)	(≥21.57 μg/L)	
Age (y)	57.0 (0.5)	56.3 (0.9)	56.1 (1.0)	56.6 (0.9)	59.1 (0.9)	0.006
Race (%)						
Non-Hispanic white	75.9 (2.1)	81.3 (3.6)	79.8 (3.2)	72.3 (3.5)	70.1 (4.4)	0.03
Non-Hispanic black	11.0 (1.2)	8.7 (2.1)	10.7 (2.2)	11.9 (2.4)	12.9 (2.1)	0.16
Other	13.1 (1.5)	10.0 (2.9)	9.5 (2.2)	15.8 (2.6)	17.0 (3.3)	0.05
High school education (%)	80.6 (1.7)	79.8 (3.9)	82.0 (4.0)	77.8 (3.8)	82.7 (3.6)	0.24
BMI (kg/m^2^)	30.4 (0.3)	30.4 (0.7)	30.1 (0.4)	30.8 (0.6)	30.1 (0.6)	0.86
Current smoking (%)	19.0 (1.7)	21.5 (4.1)	24.4 (3.8)	13.8 (3.2)	16.3 (3.7)	0.47
Serum cotinine (ng/mL)	69.4 (7.9)	90.8 (18.4)	73.9 (14.4)	56.8 (16.1)	56.0 (16.1)	0.09
Current antihypertension medication (%)	81.2 (2.4)	84.8 (4.5)	70.2 (5.2)	82.3 (3.9)	87.6 (3.3)	0.13
Systolic blood pressure (mmHg)	130.7 (1.0)	130.9 (1.3)	130.8 (1.5)	129.1 (1.8)	132.1 (2.3)	0.82
Diabetes (%)	23.2 (2.1)	20.3 (4.1)	24.6 (4.3)	23.3 (4.2)	24.7 (5.1)	0.90
Total cholesterol (mg/dL)	197.3 (2.0)	201.3 (4.9)	203.8 (4.4)	192.5 (4.0)	191.7 (4.0)	0.12
HDL cholesterol (mg/dL)	46.8 (0.7)	46.0 (1.4)	47.2 (1.6)	47.1 (1.2)	47.0 (1.5)	0.84
Predicted 10-year ASCVD risk (%) ^b^	10.8 (5.7–19.5)	10.8 (6.3–19.0)	10.0 (5.3–19.4)	9.8 (5.3–18.0)	11.7 (6.6–23.2)	0.71
Urine creatinine (mg/dL)	131.7 (3.4)	82.7 (5.6)	132.8 (7.6)	149.5 (6.3)	162.0 (6.3)	<0.001
Urine arsenic (μg/L) ^b^						
Total arsenic	9.2 (4.9–21.6)	3.1 (2.1–4.2)	7.0 (5.9–8.2)	13.2 (10.7–16.5)	39.0 (29.0–58.7)	
Dimethylarsinate	4.1 (2.6–6.9)	1.8 (1.2–2.6)	3.5 (3.0–4.5)	5.4 (4.0–7.4)	9.0 (5.7–15.1)	<0.001
Total arsenic minus arsenobetaine	6.7 (3.8–11.5)	2.3 (1.4–3.4)	5.6 (4.6–6.7)	9.2 (7.0–11.4)	17.4 (11.5–27.7)	<0.001
Arsenobetaine	1.9 (0.6–8.4)	0.7 (0.3–0.8)	0.8 (0.3–2.1)	3.7 (1.9–6.5)	20.9 (12.8–32.9)	<0.001

Abbreviations: BMI, body mass index; HDL, high-density lipoprotein; ASCVD, atherosclerotic cardiovascular disease; ^a^ adjusted for age (years) and race (non-Hispanic white, non-Hispanic black, other); ^b^ median (interquartile range). Data in other results are percentages (standard errors) for categorical variables or weighted means (standard errors) for continuous variables, unless otherwise noted.

**Table 2:** Baseline characteristics of the study population by quartiles of total arsenic concentrations in hypertensive women (*n* = 831).

Characteristics	Overall	Quartile 1	Quartile 2	Quartile 3	Quartile 4	*p* for Trend ^a^
		(≤3.30 μg/L)	(3.31–6.71 μg/L)	(6.72–14.92 μg/L)	(≥14.93 μg/L)	
Age (y)	58.6 (0.5)	58.1 (1.1)	58.9 (0.7)	60.0 (0.7)	57.4 (1.1)	0.53
Race (%)						
Non-Hispanic white	75.5 (2.2)	89.1 (2.3)	73.8 (4.0)	71.1 (3.1)	68.0 (3.9)	0.004
Non-Hispanic black	13.3 (1.6)	5.4 (1.6)	12.8 (2.4)	16.0 (2.3)	19.0 (3.0)	0.003
Other	11.2 (1.4)	5.5 (1.4)	13.4 (3.0)	12.9 (2.2)	13.0 (2.4)	0.17
High school education (%)	82.8 (1.6)	86.5 (2.7)	81.8 (3.2)	83.5 (2.4)	79.4 (3.6)	0.35
BMI (kg/m^2^)	31.9 (0.4)	30.6 (0.7)	33.4 (1.0)	32.4 (0.9)	31.4 (0.7)	0.42
Current smoking (%)	13.1 (1.5)	14.0 (3.6)	14.6 (3.3)	8.3 (2.1)	15.4 (3.6)	0.78
Serum cotinine (ng/mL)	33.7 (3.6)	45.6 (12.1)	34.8 (7.4)	21.2 (5.1)	33.2 (7.4)	0.91
Current antihypertension medication (%)	89.4 (1.4)	88.4 (3.0)	89.7 (3.2)	91.8 (2.5)	87.7 (2.5)	0.78
Systolic blood pressure (mmHg)	130.9 (0.8)	130.4 (1.4)	129.6 (1.5)	131.4 (1.8)	132.1 (1.4)	0.33
Diabetes (%)	22.3 (1.6)	24.3 (3.7)	25.9 (3.7)	20.1 (3.6)	18.8 (3.3)	0.10
Total cholesterol (mg/dL)	206.7 (1.9)	205.4 (4.1)	210.5 (3.4)	203.4 (3.5)	207.6 (3.3)	0.83
HDL cholesterol (mg/dL)	58.1 (0.8)	55.4 (1.2)	57.2 (1.8)	59.8 (1.6)	60.1 (1.8)	0.06
Predicted 10-year ASCVD risk (%) ^b^	6.3 (2.3–13.5)	5.7 (2.0–13.5)	6.6 (2.6–15.3)	6.7 (2.9–12.4)	5.9 (2.1–13.7)	0.60
Urine creatinine (mg/dL)	90.7 (3.6)	41.8 (2.2)	90.1 (5.3)	105.6 (4.4)	125.9 (9.7)	<0.001
Urine arsenic (μg/L) ^b^						
Total arsenic	6.7 (3.3–14.9)	2.1 (1.4–2.5)	5.0 (4.3–5.8)	9.2 (7.9–11.7)	26.3 (19.7–48.0)	
Dimethylarsinate	3.2 (1.9–5.4)	1.2 (1.2–1.8)	3.0 (2.2–3.6)	4.4 (3.0–5.7)	7.5 (4.4–12.2)	<0.001
Total arsenic minus arsenobetaine	4.8 (2.4–8.3)	1.4 (1.0–2.0)	4.1 (3.2–4.8)	6.6 (5.1–7.9)	15.2 (9.2–21.3)	<0.001
Arsenobetaine	1.2 (0.3–5.7)	0.3 (0.3–0.8)	0.8 (0.3–1.4)	2.5 (0.9–4.8)	12.4 (7.9–26.8)	<0.001

Abbreviations: BMI, body mass index; HDL, high-density lipoprotein; ASCVD, atherosclerotic cardiovascular disease; ^a^ adjusted for age (years) and race (non-Hispanic white, non-Hispanic black, other); ^b^ median (interquartile range). Data in other results are percentages (standard errors) for categorical variables or weighted means (standard errors) for continuous variables, unless otherwise noted.

**Table 3:** Ratio (95% CI) of predicted 10-year ASCVD risk by quartiles of urine arsenic concentrations in hypertensive men (*n* = 739).

	Quartile 1	Quartile 2	Quartile 3	Quartile 4	*p* for Trend
Total arsenic (μg/L)	≤4.92	4.93–9.20	9.21–21.56	≥21.57	
Model 1	1 (reference)	1.02 (0.80–1.30)	0.88 (0.73–1.07)	0.98 (0.76–1.25)	0.92
Model 2	1 (reference)	0.97 (0.85–1.11)	0.97 (0.86–1.09)	1.05 (0.92–1.19)	0.10
Model 3	1 (reference)	1.00 (0.88–1.14)	1.06 (0.92–1.22)	1.24 (1.02–1.53)	0.007
Dimethylarsinate (μg/L)	≤2.61	2.62–4.10	4.11–6.86	≥6.87	
Model 1	1 (reference)	0.99 (0.82–1.19)	0.94 (0.80–1.12)	0.95 (0.77–1.18)	0.64
Model 2	1 (reference)	0.97 (0.89–1.06)	0.98 (0.89–1.09)	1.07 (0.98–1.17)	0.03
Model 3	1 (reference)	0.98 (0.90–1.08)	1.01 (0.90–1.12)	1.13 (1.02–1.25)	0.003
Total arsenic minus arsenobetaine (μg/L)	≤3.83	3.84–6.66	6.67–11.51	≥11.52	
Model 1	1 (reference)	1.03 (0.84–1.27)	1.03 (0.83–1.29)	1.00 (0.78–1.27)	0.82
Model 2	1 (reference)	1.09 (0.98–1.20)	1.06 (0.95–1.20)	1.14 (1.01–1.29)	0.04
Model 3	1 (reference)	1.10 (0.99–1.22)	1.10 (0.97–1.23)	1.22 (1.05–1.40)	0.008
Arsenobetaine (μg/L)	≤0.60	0.61–1.92	1.93–8.39	≥8.40	
Model 1	1 (reference)	0.94 (0.77–1.14)	0.86 (0.73–1.03)	0.89 (0.74–1.08)	0.49
Model 2	1 (reference)	0.96 (0.88–1.06)	0.94 (0.85–1.03)	0.98 (0.89–1.07)	0.93
Model 3	1 (reference)	0.96 (0.88–1.06)	0.94 (0.86–1.03)	0.98 (0.90–1.07)	0.79

Model 1: adjusted for age (years), race (non-Hispanic white, non-Hispanic black, other) and urine creatinine (log g/L); Model 2: further adjusted for education (<high school, ≥high school), body mass index (kg/m^2^), serum cotinine (log ng/mL), diabetes (yes, no), total cholesterol (mg/dL) and high-density lipoprotein cholesterol (mg/dL); Model 3: further adjusted for arsenobetaine (log μg/L) and seafood (yes, no), except for arsenobetaine models that were further adjusted for seafood only.

**Table 4:** Ratio (95% CI) of predicted 10-year ASCVD risk by quartiles of urine arsenic concentrations in hypertensive women (*n* = 831).

	Quartile 1	Quartile 2	Quartile 3	Quartile 4	*p* for Trend
Total arsenic (μg/L)	≤3.30	3.31–6.71	6.72–14.92	≥14.93	
Model 1	1 (reference)	0.97 (0.81–1.16)	0.85 (0.70–1.04)	0.92 (0.73–1.16)	0.67
Model 2	1 (reference)	1.01 (0.90–1.14)	1.10 (0.98–1.24)	1.12 (0.97–1.29)	0.11
Model 3	1 (reference)	1.00 (0.88–1.13)	1.07 (0.92–1.24)	1.05 (0.85–1.29)	0.80
Dimethylarsinate (μg/L)	≤1.89	1.90–3.15	3.16–5.36	≥5.37	
Model 1	1 (reference)	1.00 (0.80–1.23)	1.07 (0.84–1.38)	0.94 (0.71–1.23)	0.45
Model 2	1 (reference)	1.13 (1.00–1.27)	1.14 (0.98–1.32)	1.14 (0.97–1.33)	0.39
Model 3	1 (reference)	1.11 (0.99–1.26)	1.11 (0.97–1.28)	1.10 (0.92–1.30)	0.80
Total arsenic minus arsenobetaine (μg/L)	≤2.40	2.41–4.82	4.83–8.33	≥8.34	
Model 1	1 (reference)	0.96 (0.80–1.16)	0.93 (0.72–1.21)	0.89 (0.70–1.14)	0.34
Model 2	1 (reference)	1.03 (0.92–1.16)	1.15 (0.98–1.34)	1.08 (0.94–1.25)	0.38
Model 3	1 (reference)	1.01 (0.89–1.14)	1.11 (0.94–1.31)	1.00 (0.85–1.19)	0.64
Arsenobetaine (μg/L)	≤0.30	0.31–1.16	1.17–5.70	≥5.71	
Model 1	1 (reference)	1.03 (0.86–1.24)	1.07 (0.90–1.27)	1.00 (0.84–1.19)	0.75
Model 2	1 (reference)	1.02 (0.92–1.12)	1.10 (0.99–1.21)	1.16 (1.03–1.30)	0.03
Model 3	1 (reference)	1.02 (0.92–1.13)	1.11 (1.00–1.23)	1.20 (1.05–1.37)	0.02

Model 1: adjusted for age (years), race (non-Hispanic white, non-Hispanic black, other) and urine creatinine (log g/L); Model 2: further adjusted for education (<high school, ≥high school), body mass index (kg/m^2^), serum cotinine (log ng/mL), diabetes (yes, no), total cholesterol (mg/dL) and high-density lipoprotein cholesterol (mg/dL); Model 3: further adjusted for arsenobetaine (log μg/L) and seafood (yes, no), except for arsenobetaine models that were further adjusted for seafood only.

## Supplementary Materials

The following are available online at www.mdpi.com/1660-4601/13/11/1093/s1. Figure S1: Odds ratio (95% confidence interval (CI)) of high predicted 10-year atherosclerotic cardiovascular disease (ASCVD) risk by urine arsenic concentrations in hypertensive men (*n* = 739); Figure S2: Odds ratio (95% CI) of high predicted 10-year ASCVD risk by urine arsenic concentrations in hypertensive women (*n* = 831); Table S1: Ratio (95% confidence interval (CI)) of predicted 10-year atherosclerotic cardiovascular disease (ASCVD) risk by quartiles of urine arsenic concentrations in hypertensive participants (*n* = 1570); Table S2: Ratio (95% CI) of predicted 10-year ASCVD risk by quartiles of urine arsenic concentrations in non-hypertensive participants (*n* = 1979); Table S3: Ratio (95% CI) of predicted 10-year ASCVD risk by quartiles of urine arsenic concentrations in overall participants (*n* = 3549).
