# Supplementary material for: Arsenic Exposure and Predicted 10-Year Atherosclerotic Cardiovascular Risk Using the Pooled Cohort Equations in U.S. Hypertensive Adults
Source: Int J Environ Res Public Health. 2016 Nov 7;13(11):1093. doi: 10.3390/ijerph13111093 (PMC5129303; doi:10.3390/ijerph13111093)
Supplement: Supplementary file 1 [file ijerph-13-01093-s001.pdf]

Supplementary Materials: Arsenic Exposure and Predicted 10-Year Atherosclerotic Cardiovascular Risk Using the Pooled Cohort Equations in U.S. Hypertensive Adults

Qingjiao Nong, Yiyi Zhang, Eliseo Guallar and Qiuan Zhong

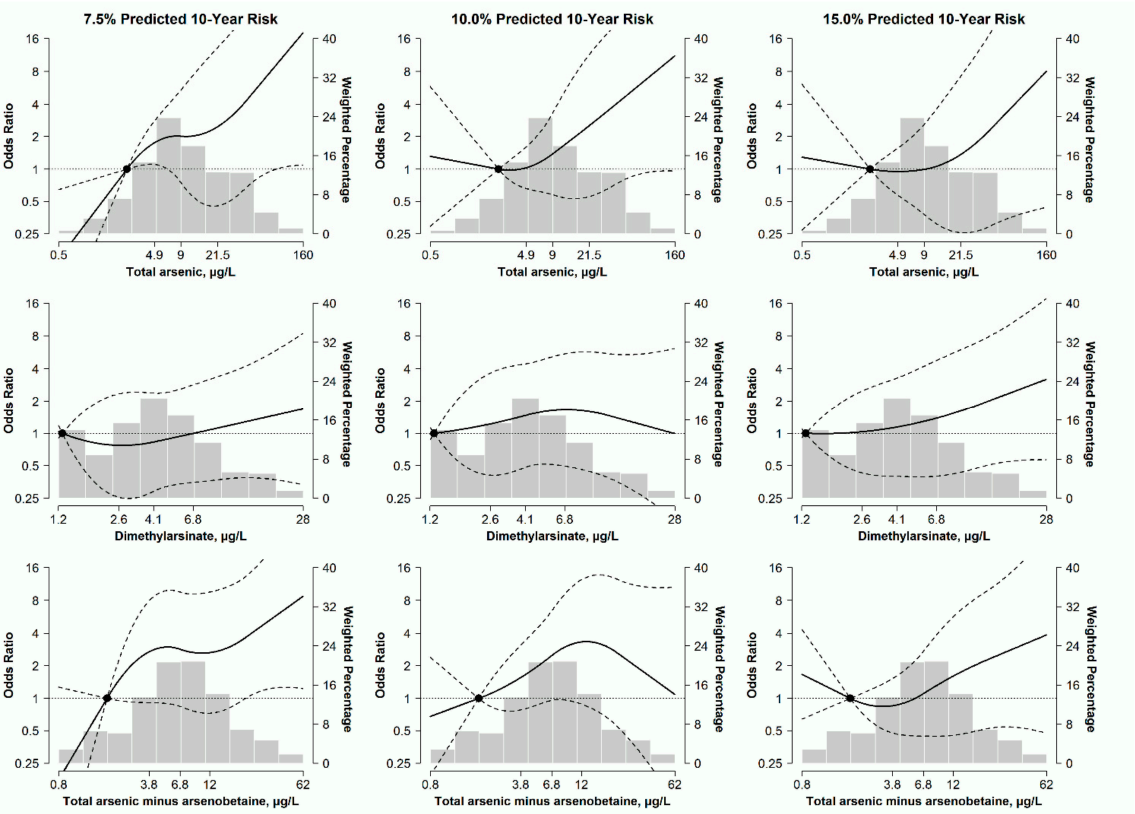

Figure S1. Odds ratio (95% confidence interval (CI)) of high predicted 10-year atherosclerotic cardiovascular disease (ASCVD) risk by urine arsenic concentrations in hypertensive men (n = 739). Odds ratios (solid lines) and 95% CIs (curved dashed lines) were based on restricted quadratic splines for log-transformed urine arsenic concentrations with knots at the 10th, 50th and 90th percentiles. The reference value (circle) was set at the 10th of each arsenical distribution. Odds ratios (95% CIs) were adjusted for age (years), race (non-Hispanic white, non-Hispanic black, other), urine creatinine (log g/L), education (<high school, ≥high school), body mass index (kg/m^2), serum cotinine (log ng/mL), diabetes (yes, no), total cholesterol (mg/dL), high-density lipoprotein cholesterol (mg/dL), arsenobetaine (log µg/L) and seafood (yes, no). Bars represent the weighted histogram of the urine arsenic distribution.

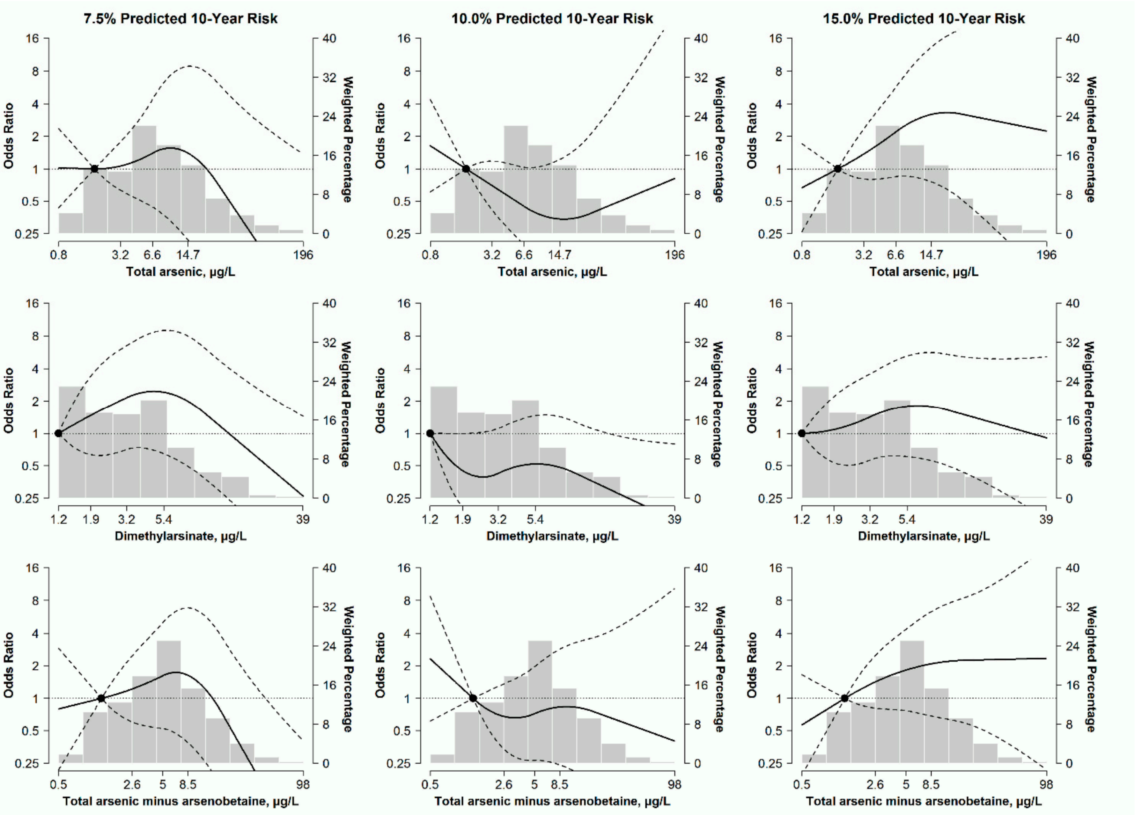

Figure S2. Odds ratio (95% CI) of high predicted 10-year ASCVD risk by urine arsenic concentrations in hypertensive women (*n* = 831). Odds ratios (solid lines) and 95% CIs (curved dashed lines) were based on restricted quadratic splines for log-transformed urine arsenic concentrations with knots at the 10th, 50th and 90th percentiles. The reference value (circle) was set at the 10th of each arsenical distribution. Odds ratios (95% CIs) were adjusted for age (years), race (non-Hispanic white, non-Hispanic black, other), urine creatinine (log g/L), education (<high school, ≥high school), body mass index (kg/m^2), serum cotinine (log ng/mL), diabetes (yes, no), total cholesterol (mg/dL), high-density lipoprotein cholesterol (mg/dL), arsenobetaine (log µg/L) and seafood (yes, no). Bars represent the weighted histogram of the urine arsenic distribution.

Table S1. Ratio (95% confidence interval (CI)) of predicted 10-year atherosclerotic cardiovascular disease (ASCVD) risk by quartiles of urine arsenic concentrations in hypertensive participants (n = 1570).

	Quartile 1	Quartile 2	Quartile 3	Quartile 4	p for Trend
Men (n = 739)					
Total arsenic (µg/L)	≤4.92	4.93–9.20	9.21–21.56	≥21.57	
Model 1	1 (reference)	1.02 (0.80–1.30)	0.88 (0.73–1.07)	0.98 (0.76–1.25)	0.92
Model 2	1 (reference)	0.97 (0.85–1.11)	0.97 (0.86–1.09)	1.05 (0.92–1.19)	0.10
Model 3	1 (reference)	1.00 (0.88–1.13)	1.05 (0.92–1.21)	1.23 (1.01–1.50)	0.008
Dimethylarsinate (µg/L)	≤2.61	2.62–4.10	4.11–6.86	≥6.87	
Model 1	1 (reference)	0.99 (0.82–1.19)	0.94 (0.80–1.12)	0.95 (0.77–1.18)	0.64
Model 2	1 (reference)	0.97 (0.89–1.06)	0.98 (0.89–1.09)	1.07 (0.98–1.17)	0.03
Model 3	1 (reference)	0.98 (0.90–1.07)	1.00 (0.90–1.11)	1.11 (1.01–1.22)	0.004
Total arsenic minus arsenobetaine (µg/L)	≤3.83	3.84–6.66	6.67–11.51	≥11.52	
Model 1	1 (reference)	1.03 (0.84–1.27)	1.03 (0.83–1.29)	1.00 (0.78–1.27)	0.82
Model 2	1 (reference)	1.09 (0.98–1.20)	1.06 (0.95–1.20)	1.14 (1.01–1.29)	0.04
Model 3	1 (reference)	1.09 (0.98–1.21)	1.09 (0.97–1.22)	1.20 (1.04–1.38)	0.01
Women (n = 831)					
Total arsenic (µg/L)	≤3.30	3.31–6.71	6.72–14.92	≥14.93	
Model 1	1 (reference)	0.97 (0.81–1.16)	0.85 (0.70–1.04)	0.92 (0.73–1.16)	0.67
Model 2	1 (reference)	1.01 (0.90–1.14)	1.10 (0.98–1.24)	1.12 (0.97–1.29)	0.11
Model 3	1 (reference)	1.00 (0.88–1.13)	1.06 (0.91–1.24)	1.04 (0.84–1.29)	0.84
Dimethylarsinate (µg/L)	≤1.89	1.90–3.15	3.16–5.36	≥5.37	
Model 1	1 (reference)	1.00 (0.80–1.23)	1.07 (0.84–1.38)	0.94 (0.71–1.23)	0.45
Model 2	1 (reference)	1.13 (1.00–1.27)	1.14 (0.98–1.32)	1.14 (0.97–1.33)	0.39
Model 3	1 (reference)	1.11 (0.98–1.25)	1.11 (0.96–1.28)	1.08 (0.91–1.28)	0.95
Total arsenic minus arsenobetaine (µg/L)	≤2.40	2.41–4.82	4.83–8.33	≥8.34	
Model 1	1 (reference)	0.96 (0.80–1.16)	0.93 (0.72–1.21)	0.89 (0.70–1.14)	0.34
Model 2	1 (reference)	1.03 (0.92–1.16)	1.15 (0.98–1.34)	1.08 (0.94–1.25)	0.38
Model 3	1 (reference)	1.00 (0.88–1.14)	1.10 (0.93–1.30)	0.99 (0.83–1.18)	0.54

Model 1: adjusted for age (years), race (non-Hispanic white, non-Hispanic black, other) and urine creatinine (log g/L); Model 2: further adjusted for education (<high school, ≥high school), body mass index (kg/m^2), serum cotinine (log ng/mL), diabetes (yes, no), total cholesterol (mg/dL) and high-density lipoprotein cholesterol (mg/dL); Model 3: further adjusted for arsenobetaine (log µg/L).

Table S2. Ratio (95% CI) of predicted 10-year ASCVD risk by quartiles of urine arsenic concentrations in non-hypertensive participants (*n* = 1979).

	Quartile 1	Quartile 2	Quartile 3	Quartile 4	*p* for Trend
Men (*n* = 990)					
Total arsenic (μg/L)	≤4.75	4.76–8.75	8.76–18.67	≥18.68	
Model 1	1 (reference)	0.92 (0.80–1.06)	0.86 (0.74–0.99)	0.84 (0.74–0.96)	0.08
Model 2	1 (reference)	1.04 (0.96–1.12)	1.04 (0.95–1.14)	1.01 (0.90–1.12)	0.69
Model 3	1 (reference)	1.03 (0.95–1.12)	1.02 (0.93–1.13)	0.97 (0.85–1.11)	0.28
Dimethylarsinate (μg/L)	≤2.32	2.33–3.95	3.96–6.14	≥6.15	
Model 1	1 (reference)	0.89 (0.76–1.04)	0.84 (0.72–0.97)	0.85 (0.72–1.01)	0.18
Model 2	1 (reference)	1.01 (0.92–1.12)	0.99 (0.90–1.10)	0.99 (0.89–1.10)	0.74
Model 3	1 (reference)	1.01 (0.91–1.12)	0.99 (0.89–1.10)	0.98 (0.88–1.10)	0.64
Total arsenic minus arsenobetaine (μg/L)	≤3.41	3.42–6.06	6.07–11.12	≥11.13	
Model 1	1 (reference)	0.80 (0.70–0.92)	0.80 (0.69–0.92)	0.81 (0.69–0.95)	0.28
Model 2	1 (reference)	0.98 (0.90–1.06)	0.97 (0.88–1.07)	0.96 (0.86–1.07)	0.55
Model 3	1 (reference)	0.98 (0.90–1.06)	0.97 (0.88–1.06)	0.94 (0.84–1.05)	0.34
Women (*n* = 989)					
Total arsenic (μg/L)	≤3.32	3.33–7.14	7.15–14.57	≥14.58	
Model 1	1 (reference)	0.86 (0.72–1.04)	0.73 (0.62–0.87)	0.67 (0.56–0 .80)	<0.001
Model 2	1 (reference)	1.04 (0.96–1.12)	0.99 (0.92–1.07)	0.97 (0.90–1.05)	0.19
Model 3	1 (reference)	1.02 (0.94–1.11)	0.96 (0.88–1.05)	0.92 (0.80–1.05)	0.11
Dimethylarsinate (μg/L)	≤1.81	1.82–3.03	3.04–5.66	≥5.67	
Model 1	1 (reference)	0.74 (0.64–0.85)	0.69 (0.57–0.84)	0.61 (0.51–0.72)	<0.001
Model 2	1 (reference)	0.99 (0.91–1.07)	1.01 (0.92–1.11)	0.95 (0.86–1.04)	0.14
Model 3	1 (reference)	0.98 (0.90–1.07)	1.01 (0.91–1.11)	0.94 (0.84–1.04)	0.10
Total arsenic minus arsenobetaine (μg/L)	≤2.33	2.34–4.79	4.80–8.95	≥8.96	
Model 1	1 (reference)	0.75 (0.64–0.87)	0.71 (0.59–0.85)	0.64 (0.53–0.78)	<0.001
Model 2	1 (reference)	0.99 (0.92–1.07)	0.99 (0.91–1.08)	0.96 (0.88–1.04)	0.24
Model 3	1 (reference)	0.99 (0.92–1.07)	0.98 (0.90–1.08)	0.95 (0.86–1.04)	0.23

Model 1: adjusted for age (years), race (non-Hispanic white, non-Hispanic black, other) and urine creatinine (log g/L); Model 2: further adjusted for education (<high school, ≥high school), body mass index (kg/m^2), serum cotinine (log ng/mL), diabetes (yes, no), total cholesterol (mg/dL), and high-density lipoprotein cholesterol (mg/dL); Model 3: further adjusted for arsenobetaine (log μg/L) and seafood (yes, no).

Table S3. Ratio (95% CI) of predicted 10-year ASCVD risk by quartiles of urine arsenic concentrations in overall participants (*n* = 3549).

	**Quartile 1**	**Quartile 2**	**Quartile 3**	**Quartile 4**	***p* for Trend**
Men (*n* = 1729)					
Total arsenic (μg/L)	≤4.78	4.79–8.96	8.97–19.42	≥19.43	
Model 1	1 (reference)	0.95 (0.83–1.09)	0.88 (0.78–0.99)	0.89 (0.78–1.03)	0.22
Model 2	1 (reference)	1.02 (0.94–1.10)	1.04 (0.95–1.13)	1.03 (0.93–1.14)	0.69
Model 3	1 (reference)	1.02 (0.95–1.11)	1.05 (0.96–1.15)	1.07 (0.95–1.20)	0.43
Dimethylarsinate (μg/L)	≤2.40	2.41–4.00	4.01–6.42	≥6.43	
Model 1	1 (reference)	0.90 (0.79–1.03)	0.87 (0.76–1.00)	0.88 (0.75–1.03)	0.27
Model 2	1 (reference)	1.01 (0.94–1.09)	1.02 (0.94–1.12)	1.05 (0.96–1.14)	0.26
Model 3	1 (reference)	1.01 (0.94–1.09)	1.03 (0.94–1.12)	1.06 (0.97–1.15)	0.15
Total arsenic minus arsenobetaine (μg/L)	≤ 3.55	3.56– 6.38	6.39–11.34	≥11.35	
Model 1	1 (reference)	0.89 (0.77–1.03)	0.91 (0.78–1.06)	0.89 (0.75–1.05)	0.42
Model 2	1 (reference)	1.04 (0.97–1.13)	1.04 (0.95–1.14)	1.06 (0.96–1.17)	0.36
Model 3	1 (reference)	1.05 (0.97–1.13)	1.04 (0.95–1.15)	1.08 (0.98–1.19)	0.22
Women (*n* = 1820)					
Total arsenic (μg/L)	≤3.31	3.32–6.90	6.91–14.74	≥14.75	
Model 1	1 (reference)	0.87 (0.74–1.01)	0.73 (0.63–0.86)	0.74 (0.62–0.88)	0.003
Model 2	1 (reference)	1.00 (0.92–1.09)	1.03 (0.94–1.13)	1.04 (0.94–1.15)	0.37
Model 3	1 (reference)	1.00 (0.91–1.08)	1.02 (0.92–1.13)	1.01 (0.88–1.17)	0.93
Dimethylarsinate (μg/L)	≤1.84	1.85–3.11	3.12–5.50	≥5.51	
Model 1	1 (reference)	0.83 (0.73–0.94)	0.85 (0.70–1.02)	0.71 (0.60–0.84)	<0.001
Model 2	1 (reference)	1.06 (0.98–1.15)	1.10 (1.00–1.21)	1.04 (0.94–1.15)	0.98
Model 3	1 (reference)	1.06 (0.98–1.14)	1.08 (0.99–1.19)	1.03 (0.93–1.14)	0.63
Total arsenic minus arsenobetaine (μg/L)	≤2.37	2.38–4.81	4.82–8.65	≥8.66	
Model 1	1 (reference)	0.81 (0.70–0.94)	0.80 (0.66–0.97)	0.70 (0.59–0.84)	<0.001
Model 2	1 (reference)	1.00 (0.93–1.08)	1.08 (0.98–1.19)	1.03 (0.94–1.12)	0.70
Model 3	1 (reference)	1.00 (0.92–1.08)	1.06 (0.96–1.18)	1.00 (0.90–1.10)	0.67

Model 1: adjusted for age (years), race (non-Hispanic white, non-Hispanic black, other) and urine creatinine (log g/L; Model 2: further adjusted for education (<high school, ≥high school), body mass index (kg/m^2), serum cotinine (log ng/mL), diabetes (yes, no), total cholesterol (mg/dL) and high-density lipoprotein cholesterol (mg/dL); Model 3: further adjusted for arsenobetaine (log μg/L) and seafood (yes, no).

© 2016 by the authors; licensee MDPI, Basel, Switzerland. This article is an open access article distributed under the terms and conditions of the Creative Commons by Attribution (CC-BY) license (http://creativecommons.org/licenses/by/4.0/).
